# The utilization of red mud waste as industrial honeycomb catalyst for selective catalytic reduction of NO

**DOI:** 10.1098/rsos.191183

**Published:** 2019-11-13

**Authors:** Lin Huangfu, Abdullahi Abubakar, Changming Li, Yunjia Li, Chao Wang, Jian Yu, Shiqiu Gao

**Affiliations:** 1 State Key Laboratory of Multi-phase Complex Systems, Institute of Process Engineering, Chinese Academy of Sciences, Beijing 100190, People's Republic of China; 2 School of Chemistry and Chemical Engineering, University of Chinese Academy of Sciences, Beijing 100049, People's Republic of China; 3 School of Chemical Engineering, Xiangtan University, Xiangtan, Hunan 411105, People's Republic of China

**Keywords:** red mud, deNO*_x_*, honeycomb catalyst, selective catalytic reduction

## Abstract

As a new way for the high-value utilization of red mud (RM) waste, we proposed an improved approach to prepare the RM-based sludge/powder via the sulfuric acid hydrothermal dissolution and NH_3_ aqueous precipitation route and then the RM-based industrial-sized honeycomb (150 × 150 × 600 mm) was successfully produced by the extrusion moulding method in pilot scale. The synthesized RM-based powdery/honeycomb catalyst exhibited more than 80% deNO*_x_* activity and good durability of H_2_O and SO_2_ above 350°C. But the decline of NO conversion was also observed above 350°C, which was confirmed to result from the increased oxygenation of NH_3_ at high temperature. To improve the NO conversion at high temperature, NH_3_ was shunted and injected into the catalyst bed at two different places (entrance and centre) to facilitate its uniform distribution, which relieved the oxidation of NH_3_ and increased deNO*_x_* efficiency with 98% NO conversion at 400°C. This work explored the industrial application feasibility for the RM-based honeycomb catalyst as well as the possible solution to decrease the oxygenation of NH_3_ at high temperature, which presented a valuable reference for the further pilot tests of RM catalyst in industry.

## 1. Introduction

Nitrogen oxides (NO*_x_*), which contribute to photochemical smog, acid rain, ozone depletion and the greenhouse effect, are the main atmospheric pollutants. Selective catalytic reduction [1,2] with NH_3_ (SCR-NH_3_) is the most promising technology for eliminating NO*_x_* emission in industry, in which catalysts play the key role. Due to the brilliant SCR activity and N_2_ selectivity at 300–400°C, commercial V_2_O_5_-WO_3_(MoO_3_)/TiO_2_ [3–6] catalysts have been widely used for decades. Nevertheless, the V-based catalysts may also encounter some inevitable disadvantages, such as the high cost as well as the toxicity of vanadium pentoxide [7]. Therefore, the development of other eco-friendly and cost-effective SCR catalysts is still the research hotspot in the field.

As the possible substitution, the low cost and non-toxicity Fe-based catalysts [8–12] have attracted much attention in recent years. Red mud (RM) is a kind of solid waste produced during the production of alumina from bauxite, which contains the metal oxides including Fe, Al, Ti, Si, Na etc. Considering its particular composition, RM may be used as Fe-based SCR catalyst for NO*_x_* removal. But its deNO*_x_* efficiency is still unsatisfactory due to its high alkalinity and low specific surface [13,14]. The previously reported deNO*_x_* efficiency of RM catalyst only achieved 31% and 40% for CO-SCR [15] and NH_3_-SCR [16], respectively. To increase the deNO*_x_* efficiency, our previous study [17] reported the preparation of RM-based powder catalyst with more than 80% NO conversion at high temperature above 350°C by a nitric acid-ball milling and neutralization-washing method. And its SCR performance was further increased after the activation of RM catalyst by SO_2_, which displayed its great application potential in industry. However, the strong volatility of HNO_3_ results in the low reaction efficiency between HNO_3_ and RM, which may be not convenient for the industrial implementation to produce the RM-based honeycomb catalyst, and the easy oxidation of NH_3_ at the temperature above 350°C may also prevent the further improvement of deNO*_x_* efficiency in industry.

In this work, we further proposed an improved approach to prepare the RM-based sludge/powder via the sulfuric acid hydrothermal dissolution and NH_3_ aqueous precipitation route, and the RM-based industrial-sized honeycomb (150 × 150 × 600 mm) was successfully produced by the extrusion moulding method in pilot scale. Moreover, the process of injecting NH_3_ in stages was proposed to decrease the oxidation of NH_3_ and increase the NO conversion at the temperature above 350°C. The results reported here move forward the utilization of RM waste as industrial honeycomb catalyst, and facilitate its further pilot test of RM-based catalyst in the real flue gas.

## 2. Experimental procedure

### 2.1. Catalyst preparations

**Figure 1.**
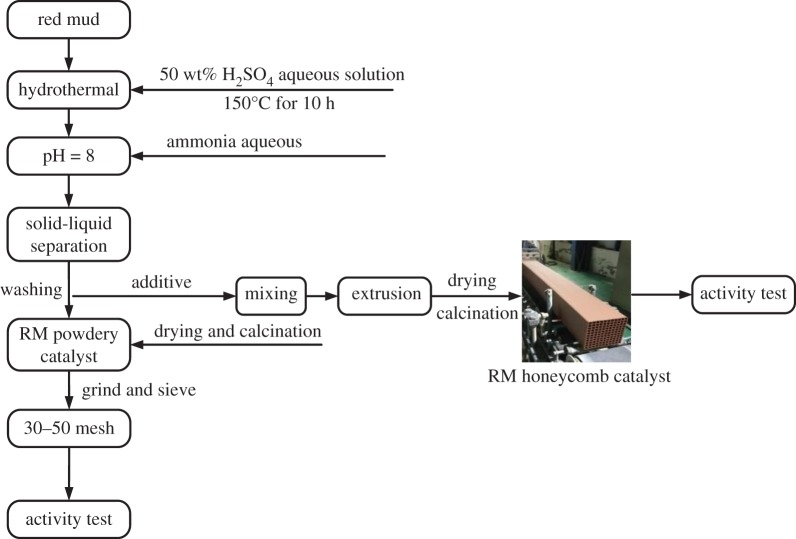
Schematic diagram for the preparation of RM catalyst/honeycomb.

The preparation process of RM powdery/honeycomb catalyst is illustrated in figure 1. Original RM (table 1A) and 50 wt% H_2_SO_4_ were mixed at a molar ratio of 1/1.2. Then the whole mixture was transferred into steel autoclave and maintained at 150°C for 10 h, during which the alkaline/alkaline-earth metal was leached, the bulk oxides such as Al_2_O_3_, Fe_2_O_3_ and TiO_2_ may also be partly dissolved by H_2_SO_4_ to form soluble aluminium sulfate, ferric sulfate and titanyl sulfate, but the SiO_2_ was nearly insoluble [16]. After the treatment by H_2_SO_4_, the composite was washed several times and neutralized to pH value of 8 with aqueous NH_3_ to remove the alkaline/alkaline-earth metal. The product of Al(OH)_3_, Fe(OH)_3_, Ti(OH)_2_ and SiO_2_ was obtained in the neutralization process. Then it was centrifuged and washed to produce the RM-based pug. The pug was either dried at 110°C and then calcined at 500°C for 3 h to prepare the powdery catalyst or was used directly to prepare the industrial-sized honeycomb catalyst (150 × 150 × 600 mm) by the extrusion moulding method. Finally, the calcined powdery sample was crushed and sieved to 30–50 mesh for SCR activity test in a fixed-bed quartz reactor (powder). The honeycomb was cut into 250 mm length with size of 30 × 30 mm.

**Table 1.** The composition information of powdery catalysts based on XRF results (wt%): (A) original RM, (B) fresh RM catalyst, (C) the sample used for 10 h, (D) the sample used for 20 h, (E) the sample used for 30 h, (F) the sample used for 40 h. Reaction condition: [NO] = [NH_3_] = 650 ppm, [O_2_] = 3 vol%, [SO_2_] = 600 ppm, [H_2_O] = 10 vol%, N_2_ balanced.

sample (%)	A	B	C	D	E	F
Fe_2_O_3_	44.18	45.13	44.50	44.96	45.52	45.34
Al_2_O_3_	19.92	15.42	15.52	15.61	15.97	15.47
TiO_2_	8.01	8.25	8.1	8.15	7.67	7.98
SiO_2_	13.43	23.69	23.37	23.16	22.79	22.81
SO_3_	0.64	6.14	6.81	6.33	6.56	7.02
Na_2_O	10.97	0.24	0.23	0.29	0.3	0.24
CaO	1.29	0.03	0.05	0.02	0.04	0.03
K_2_O	0.03	0.02	0.04	0.02	0.02	0.00

### 2.2. Catalytic performance tests

The active test of powdery catalyst was conducted in a fixed-bed quartz reactor at different space velocity with 650 ppm NO, 650 ppm NH_3_, 3 vol% O_2_, 600 ppm SO_2_ (when used), 10 vol% H_2_O (when used) and balanced with N_2_. The activity measurement of honeycomb catalyst was carried out in a deNO*_x_* square-shape steel reactor. The simulated flue gas consisted of 3 vol% O_2_, 600 ppm SO_2_ (when used), 10 vol% H_2_O (when used) and balanced with N_2_ at the ratio of NH_3_/NO = 1. The experiment was carried out in a space velocity (SV) of 6000 h^−1^ and 30 000 h^−1^, respectively. The flue gas was continually monitored by an ABB online gas analyser (ABB AO2020, Germany). Moreover, Gasmet portable FT-IR analyser (Gasmet DX4000, Finland) was used for testing the oxidation of NH_3_ at different SV.

**Figure 2.**
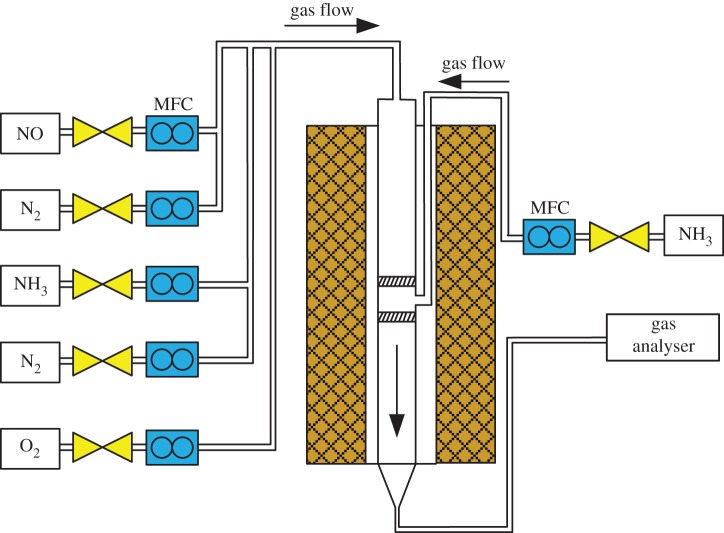
Schematic diagram of the staged injecting NH_3_ process. MFC, mass flow control.

In order to decrease the oxidation of NH_3_ and increase the NO conversion at the temperature above 350°C, the process of injecting NH_3_ in stages over RM powdery catalyst was performed. As shown in figure 2, a new powdery catalyst (30–50 mesh) test device was designed with two fixed-bed quartz reactors to inject NH_3_ at different places of catalytic bed. One part of NH_3_ was injected into the first fixed-bed quartz reactor and the other NH_3_ was directly injected into the second fixed-bed. The total mass of RM catalyst in the two fixed-bed quartz reactors is the same as the original test in the single-bed reactor, and the other reaction conditions are also identical.

The NO conversion, N_2_O yield and NH_3_ conversion were calculated according to the following equations:

2.1 $$\text{NO conversion}=\;\frac{{\left[\text{NO}\right]}_{\mathrm{in}}-\;{\left[\text{NO}\right]}_{\mathrm{out}}}{{\left[\text{NO}\right]}_{\mathrm{in}}}\times 100\text{\%},$$

2.2 $${\text{N}}_{2}\text{O}\;\text{yield}=\;{\left[{\text{N}}_{2}\text{O}\right]}_{\mathrm{out}}-{\left[{\text{N}}_{2}\text{O}\right]}_{\mathrm{in}}$$

2.3 $$\text{and}\;\;\text{N}{\text{H}}_{3}\;\text{conversion}=\;\frac{{\left[\text{N}{\text{H}}_{3}\right]}_{\mathrm{in}}-\;{\left[\text{N}{\text{H}}_{3}\right]}_{\mathrm{out}}}{{\left[\text{N}{\text{H}}_{3}\right]}_{\mathrm{in}}}\times 100\text{\%}.$$

Where [NO]_in_ and [NO]_out_ represent the concentration of gaseous NO in the inlet and outlet, respectively.

### 2.3. Catalytic characterization

The X-ray fluorescence (XRF) spectrometer (AXIOS-MAX, PANalytical BV, Holland) was used to determine the chemical composition of catalysts. X-ray diffraction (XRD) patterns were recorded on an X-ray diffractometer (Empyrean, PANalytical BV, The Netherlands) in the 2*θ* range of 5–90° with a step size of 0.4372° s^−1^ operating at 40 kV and 40 mA using Cu Kα radiation. The thermal analysis experiments were carried out in air atmosphere on a thermogravimetry/differential thermal analysis (TG/DTA 6300 produced by NSK). The mass of sample and the flow rate of air were 5 mg and 150 ml min^−1^, respectively. The heating rate was 10°C min^−1^ from 30°C to 1000°C. A nitrogen adsorption–desorption apparatus (ASAP 2020, Micromeritics Instrument Corp., USA) was used to determine the surface area and pore-size distribution of samples at 77 K. The mass of samples was 0.1 g, and each of them was degassed at 200°C for 10 h before they were measured. The morphology and microstructure of samples were recorded on a SU8020 scanning electron microscope (SEM, Hitachi, Japan). The internal microstructures of samples were observed by a JEM-2010 transmission electron microscopy (TEM, JEOL, Japan) at an accelerating voltage of 200 kV.

## 3. Results and discussion

### 3.1. Structure and morphology of powdery catalysts

**Figure 3.**
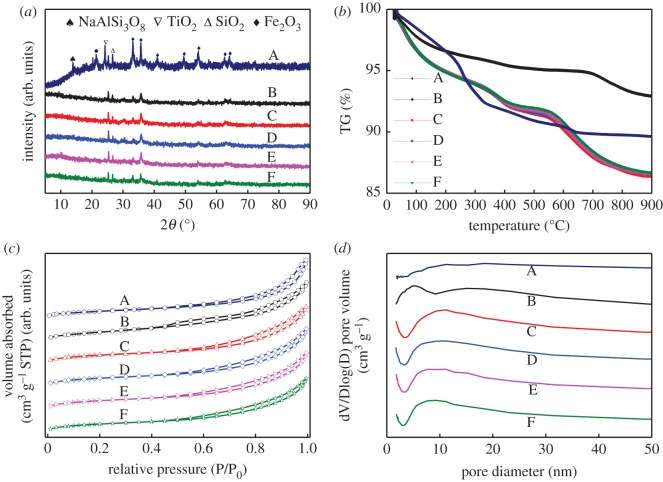
The XRD (*a*), TG (*b*), N_2_ adsorption–desorption isotherms curves (*c*) together with the corresponding pore-size distributions (*d*) patterns of: (A) original RM, (B) fresh RM catalyst, (C) the sample used for 10 h, (D) the sample used for 20 h, (E) the sample used for 30 h and (F) the sample used for 40 h. Reaction condition: [NO] = [NH_3_] = 650 ppm, [O_2_] = 3 vol%, [SO_2_] = 600 ppm, [H_2_O] = 10%, N_2_ balanced.

Table 1 summarizes the main composition of original RM, fresh RM catalyst and used RM samples (RM catalysts after SCR reaction). Due to the complexity of RM, the trace amounts of Zr, P, Ga, Cl, Ni, etc. are lower than 0.1% and not listed. The original RM (table 1A) mainly consists of Fe_2_O_3_ (44.18%), which is the active component for SCR reaction. Al_2_O_3_, TiO_2_ and SiO_2_ are common support components in catalytic system. Generally, the high Na_2_O content and other alkaline-earth metals in the original RM will interact with the major active components during catalytic reaction, resulting in the decrease in surface area and activity [13]. After the acid-hydrothermal and neutralization method, the majority of alkali/alkaline-earth metals were removed, and the fresh RM catalyst (table 1B) was obtained with the active Fe_2_O_3_ supported on SiO_2_–Al_2_O_3_–TiO_2_ composite. Figure 3*a* shows the typical XRD patterns of the synthesized RM catalysts. It can be seen that the main crystalline phases of fresh RM catalyst are α-Fe_2_O_3_, TiO_2_ and SiO_2_. The peak at 13.89° for albite disappears when compared with the original RM, which is consistent with the results of XRF. After the dissolution–precipitation process, the peak intensity Fe_2_O_3_ weakened, indicating the decreased particle size and increased dispersion of active Fe species of the obtained RM catalyst. The thermogravimetric (TG) analysis of samples is illustrated in figure 3*b*. It is generally accepted that the weight loss below 300°C belongs to the loss of adsorbed water. The second region (300–500°C) of weight loss can be attributed to the dehydroxylation for original and fresh RM samples. Besides, there is an obvious weight loss of fresh RM catalyst between 650 and 800°C, which can be assigned to the decomposition of residual sulfates.

The BET surface area measurement is also performed to have a deep insight into the change of surface area and pore-size distribution. Figure 3 illustrates the N_2_ adsorption–desorption isotherms curves (figure 3*c*) and the corresponding pore-size distribution (figure 3*d*) of catalysts, whose isotherms are close to type IV with a H_3_-type hysteresis loop, indicating the typical mesoporous characteristics. Obviously, the pore-size distribution of fresh RM catalyst (figure 3*c*-B) is broader than original RM (figure 3*c*-A). The BET surface area, pore volumes and average pore diameter are listed in table 2. It is found that the surface area of fresh RM catalyst increases significantly when compared with the original RM. The large specific surface area of RM catalyst and its small pore diameter may facilitate the dispersion and exposure of the active sites.

**Table 2.** BET surface area and pore structure results of the powdery catalysts: (A) original RM, (B) fresh RM catalyst, (C) the sample used for 10 h, (D) the sample used for 20 h, (E) the sample used for 30 h, (F) the sample used for 40 h. Reaction condition: [NO] = [NH_3_] = 650 ppm, [O_2_] = 3 vol%, [SO_2_] = 600 ppm, [H_2_O] = 10 vol%, N_2_ balanced.

catalyst	surface area (m^2^ g^−1^)	pore volume (cm^3^ g^−1^)	pore diameter (nm)
A	83	0.210	10.1
B	103	0.211	8.2
C	98	0.207	8.5
D	97	0.210	8.7
E	101	0.207	8.2
F	97	0.199	8.1

As shown in figure 3*a*, there is no obvious crystal transition/generation before and after the SCR reaction (A–E) over RM catalysts in the presence of H_2_O and SO_2_. Except for the content of SO_4_^2−^, little difference in compositions is observed between the fresh RM catalyst and catalysts after reaction according to table 1. The weight loss of the used samples (figure 3*b*) below 300°C is owing to the loss of water. The decomposition of ammonium sulfate or ammonium bisulfate [18,19] is also observed between 300 and 400°C from the residual sulfate in the used RM samples. And the weight loss at the temperature range of 400–800°C is due to the decomposition of metal sulfates. In addition, no obvious change of the BET results (figure 3*c,d*) for these used samples signifies the structure stability of our RM catalyst.

**Figure 4.**
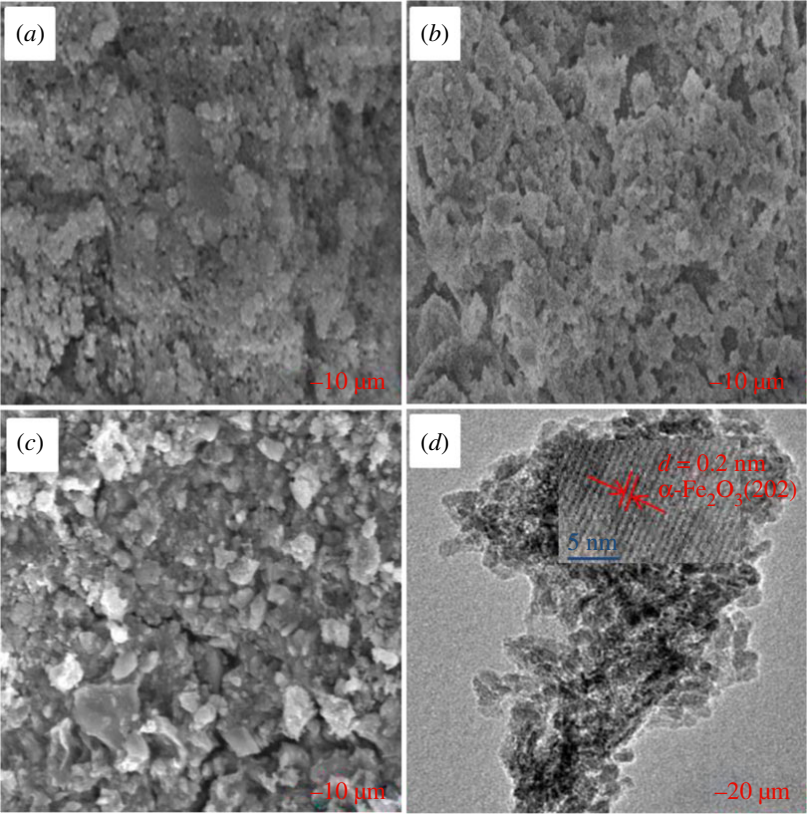
SEM images of fresh RM catalyst (*a*), RM catalyst used for 40 h (*b*) and RM honeycomb catalyst (*c*) together with the TEM image of fresh RM catalyst (*d*). Reaction condition: [NO] = [NH_3_] = 650 ppm, [O_2_] = 3 vol%, [SO_2_] = 600 ppm, [H_2_O] = 10 vol%, N_2_ balanced.

To have a better understanding of the morphology structure of RM catalyst/honeycomb, the SEM observation was carried out. In the case of fresh RM catalyst, many small irregular grains can be observed with the size of micrometre grade (figure 4*a*), and there is no significant change in morphology after SCR reaction (figure 4*b*). Figure 4*c* shows that fine RM catalyst particles on the surface of honeycomb are well dispersed. Moreover, the TEM image of fresh RM catalyst in figure 4*d* identifies the lattice fringe phase of α-Fe_2_O_3_ (2 0 2) crystal phase with the lattice distance of 0.2 nm, which is consistent with the results of XRD in figure 3*a*.

In brief, the alkali/alkaline-earth metal elements of original RM can be eliminated by acid-hydrothermal dissolution and NH_3_ aqueous neutralization method, and the as-prepared RM catalyst consists of active Fe_2_O_3_ component as well as the composite oxides of SiO_2_–Al_2_O_3_–TiO_2_. The acid and alkali treatment process also increases the specific surface area of RM catalyst, which facilitates to achieve better dispersion of the active Fe_2_O_3_ species. Moreover, no obvious difference was found between the fresh and used RM samples, indicating the good stability of our RM catalyst.

### 3.2. Catalytic performance

#### 3.2.1. The SCR activity of RM powdery catalyst

**Figure 5.**
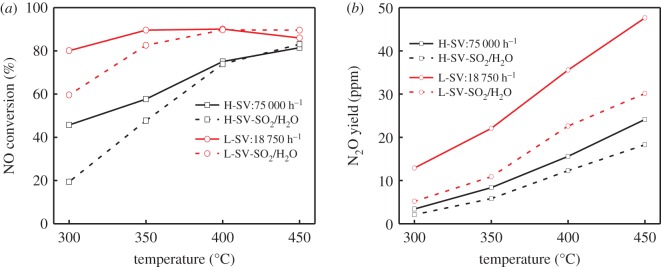
The NO conversion (*a*) and N_2_O yield (*b*) with/without H_2_O and SO_2_ over powdery catalysts. Reaction condition: [NO] = [NH_3_] = 650 ppm, [O_2_] = 3 vol%, [SO_2_] = 600 ppm, [H_2_O] = 10 vol%, N_2_ balanced.

The NO conversion of RM powdery catalyst is investigated and illustrated in figure 5*a*. The temperature window for above 80% NO conversion ranged from 350 to 450°C (SV = 18 750 h^−1^) in the absence of H_2_O and SO_2_, which was much higher than that of 31 and 40% deNO*_x_* efficiency in the previous study [15,16]. The lower the SV, the longer the contact time of the gas and catalyst, resulting in the increase of NO conversion. But the produced N_2_O is also increased at low SV. The decrease in deNO*_x_* activity was observed for the addition of H_2_O and SO_2_ at low temperature below 350°C. However, as the temperature increased, the inhibition effect of H_2_O and SO_2_ decreased. Particularly, H_2_O and SO_2_ slightly promoted the NO conversion of the RM catalyst above 400°C. Meanwhile, it can be found in figure 5*b* that the N_2_O formation decreased obviously in the presence of H_2_O and SO_2_, especially at the low SV and high temperature. Generally, H_2_O and SO_2_ may damage the low-temperature SCR activity, apparently due to the blocking of the active sites by ammonium sulfate [20] and the competitive adsorption between H_2_O and NH_3_ [21,22]. For this reason, the N_2_O formation also decreased. As the temperature increased, the H_2_O could be easily desorbed from inactive Fe sites, resulting in the recovery of Fe active sites as well as the NO conversion. Besides, H_2_O could inhibit the oxidation of NH_3_ at high temperature, which is of benefit to the improvement of SCR activity above 400°C. The observed effect of H_2_O and SO_2_ on the deNO*_x_* activity of RM catalysts is consistent with these previous reports [17], and the RM catalyst may also have good stability and resistance to H_2_O and SO_2_ above 350°C.

#### 3.2.2. SCR performance of RM honeycomb catalyst

**Figure 6.**
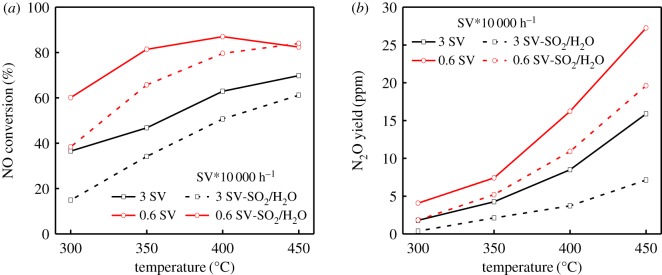
The NO conversion (*a*) and N_2_O yield (*b*) with/without H_2_O and SO_2_ over RM honeycomb catalyst. Reaction condition: [NO] = [NH_3_] = 650 ppm, [O_2_] = 3 vol%, [SO_2_] = 600 ppm, [H_2_O] = 10 vol%, N_2_ balanced, velocity = 3 N m s^−1^.

To explore the application feasibility of RM catalyst in industry, the RM honeycomb catalyst was further produced by extrusion moulding method. As shown in figure 6*a*, the NO conversion increased along with the increasing temperature at high SV (30 000 h^−1^), but the best NO conversion was only 60%. To further increase the deNO*_x_* efficiency, the experiment at low SV was conducted. The RM honeycomb catalyst achieved more than 80% NO conversion in the temperature range of 350–450°C at 6000 h^−1^, and the maximum NO conversion reached 87% at 400°C. With the temperature further increased, the NO conversion declined while the formation of N_2_O (figure 6*b*) increased dramatically. Additionally, on the basis of the fact that the exhaust usually contains water vapour and a certain amount of SO_2_, the effects of H_2_O and SO_2_ on the catalytic performance of RM honeycomb catalysts were further investigated. Although the addition of 10% H_2_O and 600 ppm SO_2_ significantly suppressed the NO conversion at low temperature, the adverse effect progressively weakened with increased temperature. Meanwhile, the N_2_O formation was lower in the presence of H_2_O and SO_2_, and best NO conversion could reach above 80% at 450°C with the N_2_O lower than 20 ppm on the condition. The SCR performance of our RM honeycomb catalyst is in good agreement of the results of RM powdery catalyst, demonstrating that it is feasible to use RM-based catalyst for denitrification in industrial applications.

### 3.3. The effect of ammoxidation on SCR efficiency at the temperature above 350°C

#### 3.3.1. The features of ammoxidation during the SCR reaction over RM catalysts

**Figure 7.**
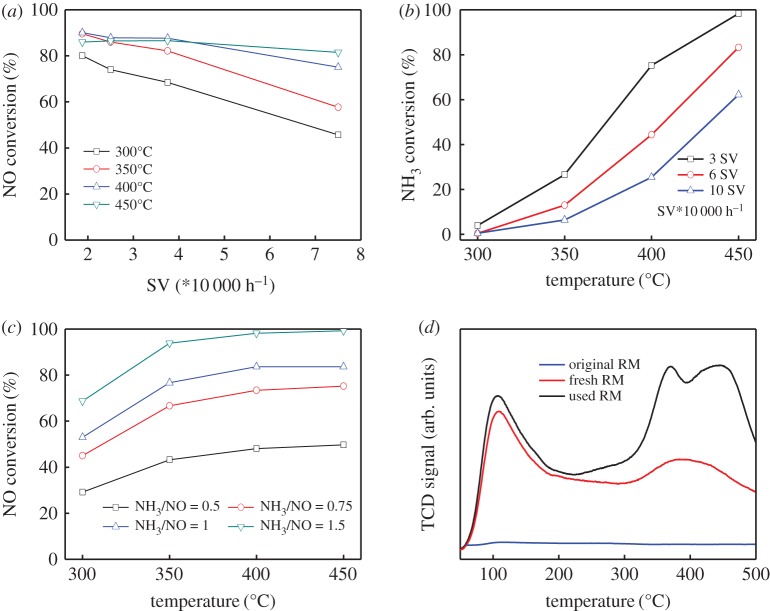
The effect of space velocity (*a*), NH_3_ oxidation (*b*) and NH_3_/NO ratio (*c*) together with the result of NH_3_-TPD (*d*) over RM catalyst. Reaction condition: [NH_3_] = 650 ppm, [NO] = 325–975 ppm, [O_2_] = 3 vol%, N_2_ balanced.

As stated above, it can be concluded that for both the powder and honeycomb RM catalyst, the deNO*_x_* efficiency is superior at low SV than that at high SV. Nevertheless, more N_2_O was formed at low SV with low SCR selectivity, which prevented the further improvement of deNO*_x_* efficiency close to 100% at high temperature above 350°C. Therefore, more tests were done to reveal the features of ammoxidation during the SCR reaction. Figure 7*a* illustrates the effect of SV on SCR performance over powdery RM catalyst. It displayed that deNO*_x_* efficiency increased linearly and apparently at 300–350°C with the decrease in SV. But at high reaction temperature above 350°C, the decrease in SV had no obvious improvement on deNO*_x_* efficiency. The NO conversion was still lower than 90% even at low SV and high temperature, which was different from V-based catalyst with almost 100% deNO*_x_* efficiency on proper condition. Most interestingly, the NO conversion of 450°C even decreased at lower SV, implying that it is impossible to improve the high-temperature SCR activity by simply increasing the amount of catalyst.

The NH_3_ oxidation measurement at different SV is observed in figure 7*b*. It exhibited that the NH_3_ oxidation drastically increased above 350°C. As the SV decreased, the oxidation of NH_3_ enhanced. Especially at 450°C, NH_3_ was almost completely oxidized at the SV of 30 000 h^−1^ due to the longer contact time between catalyst and NH_3_. Although NO conversion has been improved with the decrease in SV, the oxidation of NH_3_ at low SV was much higher than that at high SV in the high-temperature range. The oxidation of NH_3_ decreased the supply of NH_3_ for the SCR reaction and thus prevented the achieved high NO conversion at low SV and high temperature for RM catalysts.

To further confirm this point, we investigated the effect of NH_3_/NO ratio and the result is depicted in figure 7*c*. It can be seen that NO conversion increased with the increase in NH_3_/NO ratio. And it reached the theoretical value at 400°C when the concentration of NH_3_ was lower than NO. However, only 80% NO conversion was obtained when NH_3_/NO was 1 and it achieved almost 100% NO conversion at the ratio of NH_3_/NO = 1.5. Thus, it can be deduced that when NH_3_ was sufficient such as the NH_3_/NO ≥ 1, a large amount of NH_3_ was oxidized and consumed at high temperature. As a contrast, the low ratio of NH_3_/NO showed the low oxygenation efficiency of NH_3_ with its high utilization efficiency. The results were also consistent with previous reports about the competition reaction between NH_3_-SCR and NH_3_-SCO (NH_3_-selective catalytic oxidation) [8,23,24]. The -NH_2_ intermediate species [25,26] activated by NH_3_ preferentially reacted with NO at the lower NH_3_ concentration. With the increase in NH_3_/NO ratio, the oxidation of NH_3_ was enhanced, resulting in the lack of NH_3_ to react with NO. Accordingly, the maximum theoretical value of NO conversion cannot be reached owing to the severe oxidation of NH_3_ at high-temperature range.

The oxidizability of NH_3_ is related to the acid sites of RM catalysts, thus NH_3_-TPD (temperature programmed desorption) test is carried out as profiled in figure 7*d*. It can be seen that the adsorption capacity of NH_3_ increased significantly for the fresh RM sample compared with the original one due to the elimination of alkali/alkaline-earth metals. The desorption peak centred at about 100°C can be assigned to physically adsorbed NH_3_. Another obvious desorption peak can be observed from 300 to 500°C, which may be due to the desorption of ionic NH_4_^+^ bound to strong Brønsted acid sites and coordinated NH_3_ bound to Lewis acid sites. It was reported [17,27] that NH_3_ was mainly absorbed on the Brönsted acid sites at relatively low temperature to form NH_4_^+^ and then react with NO to generate N_2_ and H_2_O, while the coordinated NH_3_ bound to Lewis acid sites usually response for the SCR activity at high temperature. Both of the Brönsted acid sites and Lewis acid sites are important in the SCR/SCO reaction with NH_3_. As for the used RM catalyst, the residual sulfate may further increase the NH_3_ adsorption. These strong acid sites between 300 and 500°C will facilitate the strong adsorption of ammonia and will be also beneficial for the NH_3_-SCO reaction, resulting in the decreased SCR selectivity. All of these results indicate that the severe oxidation of NH_3_ resulted in the low actual ratio of NH_3_/NO and thus low reaction efficiency of SCR.

#### 3.3.2. The improved deNO*_x_* efficiency by injecting NH_3_ in stages

**Figure 8.**
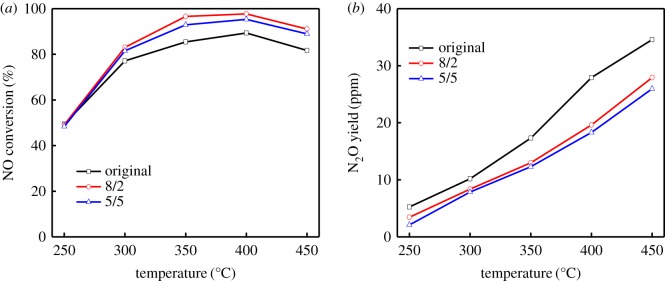
Comparison of NO conversion (*a*) and N_2_O yield (*b*) between the original single-bed process and the process of injecting NH_3_ in stages. Reaction condition: [NH_3_] = 650 ppm, [NO] = 650 ppm, [O_2_] = 3 vol%, N_2_ balanced, SV = 30 000 h^−1^.

In order to decrease the oxidation of NH_3_ and increase the NO conversion at high temperature, the process of injecting NH_3_ in stages over RM powdery catalyst was proposed. The effects of NH_3_ concentration and distribution on the SCR reaction are illustrated in figure 8. Despite that the NO conversion also decreased with increasing temperature above 400°C, the new process demonstrated much more advantages in NO conversion than the original single-bed reactor (the black line). The NO conversion increased from 89 to 95% at 400°C after the introduction of injecting NH_3_ in stages at 5/5 NH_3_ distribution of the two parts. Particularly, the best NO conversion achieved 98% when the NH_3_ distribution of the two parts is 8/2, which is also coincident with the result in figure 7*c*. Meanwhile, it is worth noting that the N_2_O formation of our new process was much lower in the entire temperature range, which indicated the decrease in NH_3_ oxidation with the improved N_2_ selectivity. These results demonstrated that the injection of NH_3_ in stages can significantly weaken the oxidation of NH_3_ at high temperature, increase the utilization of NH_3_ and improve the high-temperature deNO*_x_* efficiency. According to our experimental results, the NH_3_ distribution of 8/2 for the two parts may be a suitable choice, and the process of injecting NH_3_ in stages may facilitate to reach high deNO*_x_* efficiency at high temperature by reducing oxidation of NH_3_ for the further application of RM catalysts in industry.

## 4. Conclusion

The RM-based powdery/honeycomb catalyst was successfully produced through the improved H_2_SO_4_-NH_3_ pretreatment and subsequent extrusion process with above 80% deNO*_x_* activity and good durability of H_2_O and SO_2_ above 350°C. The characterization results of XRF, XRD and BET indicated that the elimination of alkali/alkaline-earth metal elements from original RM increased the specific surface area and facilitated to achieve better dispersion of the active components of our RM-based catalyst. Although NO conversion increased with the decrease in SV, the theoretical 100% NO conversion still cannot be obtained even at low SV. The further test of SV, NH_3_/NO ratio, oxygenation efficiency of NH_3_ as well as the NH_3_-TPD confirmed that the severe oxidation of NH_3_ over the strong acid sites of RM catalyst resulted in the low actual ratio of NH_3_/NO at high temperature, and the lack of NH_3_ prevented the achievement of ideal SCR reaction efficiency in theory. As an effective solution, the process of injecting NH_3_ in stages was carried out and could significantly reduce the oxidation of NH_3_ owing to the more reasonable distribution of NH_3_ in the SCR reactor. The best NO conversion increased to 98% when the distribution of NH_3_ is 8/2 in the two parts. The successful production of RM-based industrial-sized honeycomb catalyst as well as the improved SCR reaction efficiency by the process of injecting NH_3_ in stages signify the application feasibility for the utilization of RM waste as industrial honeycomb deNO*_x_* catalyst.

## Supplementary Material

Data of experiments

Reviewer comments

## Supplementary Material

Data of characterizations

## Data accessibility

The data that support the findings of this study are openly available from electronic supplementary material, which includes the raw data of the experiments and characterizations.

## References

[1] Benson SA, Laumb JD, Crocker CR, Pavlish JH 2005 SCR catalyst performance in flue gases derived from subbituminous and lignite coals. Fuel Process. Technol. 86, 577–613. (10.1016/j.fuproc.2004.004)

[2] Xiang J, Wang PY, Su S, Zhang LQ, Cao F, Sun ZJ, Xiao X, Sun LS, Hu S 2015 Control of NO and Hg-0 emissions by SCR catalysts from coal-fired boiler. Fuel Process. Technol. 135, 168–173. (10.1016/j.fuproc.2014.12.044)

[3] Alemany LJ, Berti F, Busca G, Ramis G, Robba D, Toledo GP, Trombetta M 1996 Characterization and composition of commercial V_2_O_5_-WO_3_-TiO_2_ SCR catalysts. Appl. Catal. B-Environ. 10, 299–311. (10.1016/s0926-3373(96)00032-x)

[4] Forzatti P, Nova I, Tronconi E, Kustov A, Thogersen JR 2012 Effect of operating variables on the enhanced SCR reaction over a commercial V_2_O_5_-WO_3_/TiO_2_ catalyst for stationary applications. Catal. Today 184, 153–159. (10.1016/cattod.2011.11.006)

[5] Qiu Y, Liu B, Du J, Tang Q, Liu ZH, Liu RL, Tao CY. 2016 The monolithic cordierite supported V_2_O_5_-MoO_3_/TiO_2_ catalyst for NH_3_-SCR. Chem. Eng. J. 294, 264–272. (10.1016/j.cej.2016.02.094)

[6] Zhao K, Han WL, Tang ZC, Zhang GD, Lu JY, Lu GX, Zhen XP 2016 Investigation of coating technology and catalytic performance over monolithic V_2_O_5_-WO_3_/TiO_2_ catalyst for selective catalytic reduction of NO*_x_* with NH_3_. Colloid Surf. A-Physicochem. Eng. Asp. 503, 53–60. (10.1016/j.colsurfa.2016.05.014)

[7] Busca G, Lietti L, Ramis G, Berti F 1998 Chemical and mechanistic aspects of the selective catalytic reduction of NO*_x_* by ammonia over oxide catalysts: a review. Appl. Catal. B-Environ. 18, 1–36. (10.1016/S0926-3373(98)00040-X)

[8] Fabrizioli P, Burgi T, Baiker A 2002 Environmental catalysis on iron oxide-silica aerogels: selective oxidation of NH_3_ and reduction of NO by NH_3_. J. Catal. 206, 143–154. (10.1006/jcat.2001.3475)

[9] Yang S et al. 2012 Fe-Ti spinel for the selective catalytic reduction of NO with NH_3_: mechanism and structure–activity relationship. Appl. Catal. B-Environ. 117, 73–80. (10.1016/j.apcatb.2012.01.001)

[10] Jiang SY, Zhou RX 2015 Ce doping effect on performance of the Fe/beta catalyst for NO*_x_* reduction by NH_3_. Fuel Process. Technol. 133, 220–226. (10.1016/j.fuproc.2015.02.004)

[11] Qu ZP, Miao L, Wang H, Fu Q 2015 Highly dispersed Fe_2_O_3_ on carbon nanotubes for low-temperature selective catalytic reduction of NO with NH_3_. Chem. Commun. 51, 956–958. (10.1039/c4cc06941b) 25434305

[12] Wei Y, Chen YL, Wang R 2018 Rare earth salt of 12-tungstophosphoric acid supported on iron oxide as a catalyst for selective catalytic reduction of NO*_x_*. Fuel Process. Technol. 178, 262–270. (10.1016/j.fuproc.2018.06.001)

[13] Ordonez S 2008 Comments on ‘Catalytic applications of red mud, an aluminium industry waste: a review’. Appl. Catal. B-Environ. 84, 732–733. (10.1016/j.apcatb.2008.06.001)

[14] Wang XK, Zhang YH, Lv FZ, An Q, Lu RR, Hu P, Jiang SB 2015 Removal of alkali in the red mud by SO_2_ and simulated flue gas under mild conditions. Environ. Prog. Sustain. Energy 34, 81–87. (10.1002/ep.11958)

[15] Mohapatro S, Rajanikanth BS 2012 Dielectric barrier discharge cascaded with red mud waste to enhance NO*_x_* removal from diesel engine exhaust. IEEE Trans. Dielectr. Electr. Insul. 19, 641–647. (10.1109/tdei.2012.6180259)

[16] Sushil S, Batra VS 2008 Catalytic applications of red mud, an aluminium industry waste: a review. Appl. Catal. B-Environ. 81, 64–77. (10.1016/j.apcatb.2007.12.002)

[17] Li CM, Zeng H, Liu PL, Yu J, Guo F, Xu GW, Zhang ZG 2017 The recycle of red mud as excellent SCR catalyst for removal of NO*_x_*. RSC Adv. 7, 53 622–53 630. (10.1039/c7ra10348d)

[18] Shi YJ, Shu H, Zhang YH, Fan HM, Zhang YP, Yang LJ 2016 Formation and decomposition of NH_4_HSO_4_ during selective catalytic reduction of NO with NH_3_ over V_2_O_5_-WO_3_/TiO_2_ catalysts. Fuel Process. Technol. 150, 141–147. (10.1016/j.fuproc.2016.05.016)

[19] Ye D, Qu RY, Song H, Gao X, Luo ZY, Ni MJ, Cen KF 2016 New insights into the various decomposition and reactivity behaviors of NH_4_HSO_4_ with NO on V_2_O_5_/TiO_2_ catalyst surfaces. Chem. Eng. J. 283, 846–854. (10.1016/j.cej.2015.08.020)

[20] Wang XB, Gui KT 2013 Fe_2_O_3_ particles as superior catalysts for low temperature selective catalytic reduction of NO with NH_3_. J. Environ. Sci. 25, 2469–2475. (10.1016/S1001-0742(12)60331-3) 24649679

[21] Willey RJ, Eldridge JW, Kittrell JR 1985 Mechanistic model of the selective catalytic reduction of nitric-oxide with ammonia. Ind. Eng. Chem. Prod. Res. Dev. 24, 226–233. (10.1021/i300018a011)

[22] Yang SJ et al. 2012 A novel magnetic Fe-Ti-V spinel catalyst for the selective catalytic reduction of NO with NH_3_ in a broad temperature range. Catal. Sci. Technol. 2, 915–917. (10.1039/c2cy00459c)

[23] Long RQ, Yang RT 2002 Selective catalytic oxidation of ammonia to nitrogen over Fe_2_O_3_-TiO_2_ prepared with a sol-gel method. J. Catal. 207, 158–165. (10.1006/jcat.2002.3545)

[24] Yang SJ, Liu CX, Chang HZ, Ma L, Qu Z, Yan NQ, Wang CZ, Li JH 2013 Improvement of the activity of gamma-Fe_2_O_3_ for the selective catalytic reduction of NO with NH_3_ at high temperatures: NO reduction versus NH_3_ oxidization. Ind. Eng. Chem. Res. 52, 5601–5610. (10.1021/ie303272u)

[25] Shu Y, Wang HC, Zhu JW, Zhang F 2014 Mechanism of the selective catalytic reduction of NO*_x_* with NH_3_ over W-doped Fe/TiO_2_ catalyst. Chem. Res. Chin. Univ. 30, 1005–1010. (10.1007/s40242-014-4161-4)

[26] Liang H, Gui K, Zha XB 2016 Drifts study of gamma Fe_2_O_3_ nano-catalyst for low-temperature selective catalytic reduction of NO*_x_* with NH_3_. Can. J. Chem. Eng. 94, 1668–1675. (10.1002/cjce.22546)

[27] Wang T, Wan ZT, Yang XC, Zhang XY, Niu XX, Sun BM 2018 Promotional effect of iron modification on the catalytic properties of Mn-Fe/ZSM-5 catalysts in the Fast SCR reaction. Fuel Process. Technol. 169, 112–121. (10.1016/j.fuproc.2017.09.029)

